# Real-time, random-access organ screening for carbapenem-resistant organisms (CRO) reduces CRO-associated, donor-derived infection mortality in lung transplant recipients

**DOI:** 10.1007/s15010-023-02089-6

**Published:** 2023-08-31

**Authors:** Wen-Yong Zhou, Lei Shen, Jian-Xin Shi, Xing-Hui Gao, Jun Yang, Shi-Jie Fu, Xu-Feng Pan, Min-Fang Zhu, Shen Zhang, Chong Zhang, Feng Li, Hai Zhang, Feng Yao, Fred C. Tenover, Yi-Wei Tang, Wen-Tao Fang

**Affiliations:** 1 Department of Thoracic Surgery, Shanghai Chest Hospital, Shanghai Jiao Tong University School of Medicine, Shanghai, China; 2 Shanghai Institute of Immunology, Department of Immunology and Microbiology, and Key Laboratory of Cell Differentiation and Apoptosis of the Chinese Ministry of Education, Shanghai Jiao Tong University School of Medicine, Shanghai, China; 3 Shanghai Key Laboratory of Tumor Microenvironment and Inflammation, Shanghai Jiao Tong University School of Medicine, Shanghai, China; 4 Medical Affairs, Danaher Diagnostic Platform/Cepheid, Shanghai, China; 5 Department of Critical Care Medicine, Shanghai Chest Hospital, Shanghai Jiao Tong University School of Medicine, Shanghai, China; 6 Department of Pulmonary Medicine, Shanghai Chest Hospital, Shanghai Jiao Tong University School of Medicine, Shanghai, China; 7 Medical and Scientific Affairs, Cepheid, Sunnyvale, CA USA

**Keywords:** Donor-derived infection, Carbapenem-resistant organisms, Lung transplantation, Point-of-care testing, Prognosis

## Abstract

**Purpose:**

Donor-derived infection (DDI) has become an important factor affecting the prognosis of lung transplantation patients. The risks versus benefits of using donor organs infected with multidrug-resistant organisms (MDRO), especially carbapenem-resistant organisms (CRO), are frequently debated. Traditional microbial culture and antimicrobial susceptibility testing at present fail to meet the needs of quick CRO determination for donor lungs before acquisition. In this study, we explored a novel screening method by using Xpert^®^ Carba-R assay for CRO in donor lungs in a real-time manner to reduce CRO-associated DDI mortality.

**Methods:**

This study was registered on chictr.org.cn (ChiCTR2100053687) on November 2021. In the Xpert Carba-R screening group, donor lungs were screened for CRO infection by the Xpert Carba-R test on bronchoalveolar fluid (BALF) before acquisition. If the result was negative, donor lung acquisition and subsequent lung transplantation were performed. In the thirty-five potential donors, nine (25.71%) with positive Xpert Carba-R results in BALF were declined for lung transplantation. Twenty-six recipients and the matching CRO-negative donor lungs (74.29%) were included in the Xpert Carba-R screening group. In the control group, nineteen recipients underwent lung transplants without Xpert Carba-R screening. The incidence and mortality of CRO-associated DDI were collected and contrasted between the two groups.

**Results:**

Multivariate analysis showed that CRO-related death due to DDI within 60 days was significantly lower in the Xpert Carba-R screening group than that in the control group (OR = 0.05, 95% CI 0.003–0.74, *p* = 0.03).

**Conclusion:**

Real-time CRO screening of donor lungs before transplantation at the point of care by the Xpert Carba-R helps clinicians formulate lung transplantation strategies quickly and reduces the risk of subsequent CRO infection improving the prognosis of lung transplantation.

## Introduction

Lung transplantation is the only effective treatment option for patients with end-stage lung disease. Lung donations come mainly from patients who have suffered brain death (i.e., donation after brain death, DBD) and donation after cardiac death (DCD) [1]. Infection is one of the leading causes of both short-term and long-term death in lung transplantation recipients [1, 2]. Donor-derived infection (DDI) is an important infection in lung transplant recipients [3]. Although the epidemiology varies markedly among different geographical regions, risk factors of prolonged (> 7 days) ICU stay, vasopressor support, and cardiopulmonary resuscitation increases colonization with multidrug-resistant organisms (MDRO) including extended-spectrum β-lactamase -producing Enterobacteriaceae (ESBL-E) and carbapenem-resistant organisms (CRO) in donors [4]. The current study suggests that MDRO or CRO may significantly contribute to the burden of bacterial DDI [5, 6]. Lung transplant recipients appear to be disproportionately affected, potentially due to the high frequency of MDRO colonization of the donor respiratory tract [7, 8]. MDRO-associated DDI or CRO-associated DDI may play a role in the development of early posttransplant infections in transplant recipients [9], leading to several complications, including stomal leak, bleeding, graft loss, and even death [7, 10, 11]. If donor colonization or infection with CRO is known before transplantation, a risk–benefit evaluation should be made based on the organ to be transplanted and the site of the positive donor cultures. Clinicians suggest that acquiring the lung from a donor experiencing CRO bacteremia or respiratory colonization for transplantation should be avoided [3].

Traditionally, CRO screening of donor lungs is based on bacterial culture followed by phenotypic antimicrobial susceptibility testing. However, there are two main problems with culture-based screening: (i) the time to results, which requires at least 72 h, is too slow to meet the needs of donor lung acquisition; and (ii) the requirements for trained personnel, equipment, sites, etc., which are inconsistent with the organ procurement organizations (OPOs) [12]. The culture method has a result reporting time delay, which may lead to occult CRO infection in the donor lungs during the period before culture and susceptibility test results are available. Therefore, a rapid, simple, and accurate technique for CRO screening of donor organs is urgently needed at the point of care. This could effectively reduce latent donor CRO infection, reduce the incidence of postoperative DDI and infection-related adverse events of lung transplantation, and improve the prognosis of patients who receive lung transplantation.

The Xpert^®^ Carba-R assay (Cepheid, Sunnyvale, CA, USA) is an on-demand PCR test specifically designed for the qualitative detection and differentiation of five common carbapenemase gene families, including *bla*_*KPC*_*, bla*_*NDM*_*, bla*_*VIM*_*, bla*_*IMP*_, and *bla*_*OXA-48*_ with results available in less than one hour [13]. Due to the portability of the instrument, the donor lung acquisition team can test the donor lung for CRO at the OPO and decide whether to acquire the lung and perform the transplantation after obtaining the CRO results in real time. This approach would help avoid the acquisition of a CRO-infected donor lung and reduce the occurrence of CRO-associated DDI and infection-related complications after lung transplantation caused by using CRO-infected lungs. This study used the GeneXpert II instrument combined with the Xpert Carba-R test on-site to detect CRO in donor bronchoalveolar fluid (BALF) as an off-label specimen before donor lung acquisition.

## Materials and methods

### Patients and study design

This historically controlled prospective trial was approved by the Ethics Committee of Shanghai Chest Hospital, Shanghai Jiao Tong University (No. IS21101) and registered on chictr.org.cn (ChiCTR2100053687). Written informed consent was obtained from the recipients. We obtained the authorization of the donor's next of kin and the Ethics Committee to use donor-derived biological samples for clinical and research purposes. All recipients who enrolled in the study accepted lung transplant treatment at Shanghai Chest Hospital. The screening group included patients who underwent lung transplantation from November 2021 to September 2022 with negative CRO results of the donor lung by the Xpert Carba-R test. The historically controlled group included patients who underwent lung transplantation from January 2016 to December 2020. All patients who were included in the Xpert Carba-R screening group and control group met the same inclusion criteria of this study.

**Fig. 1:**
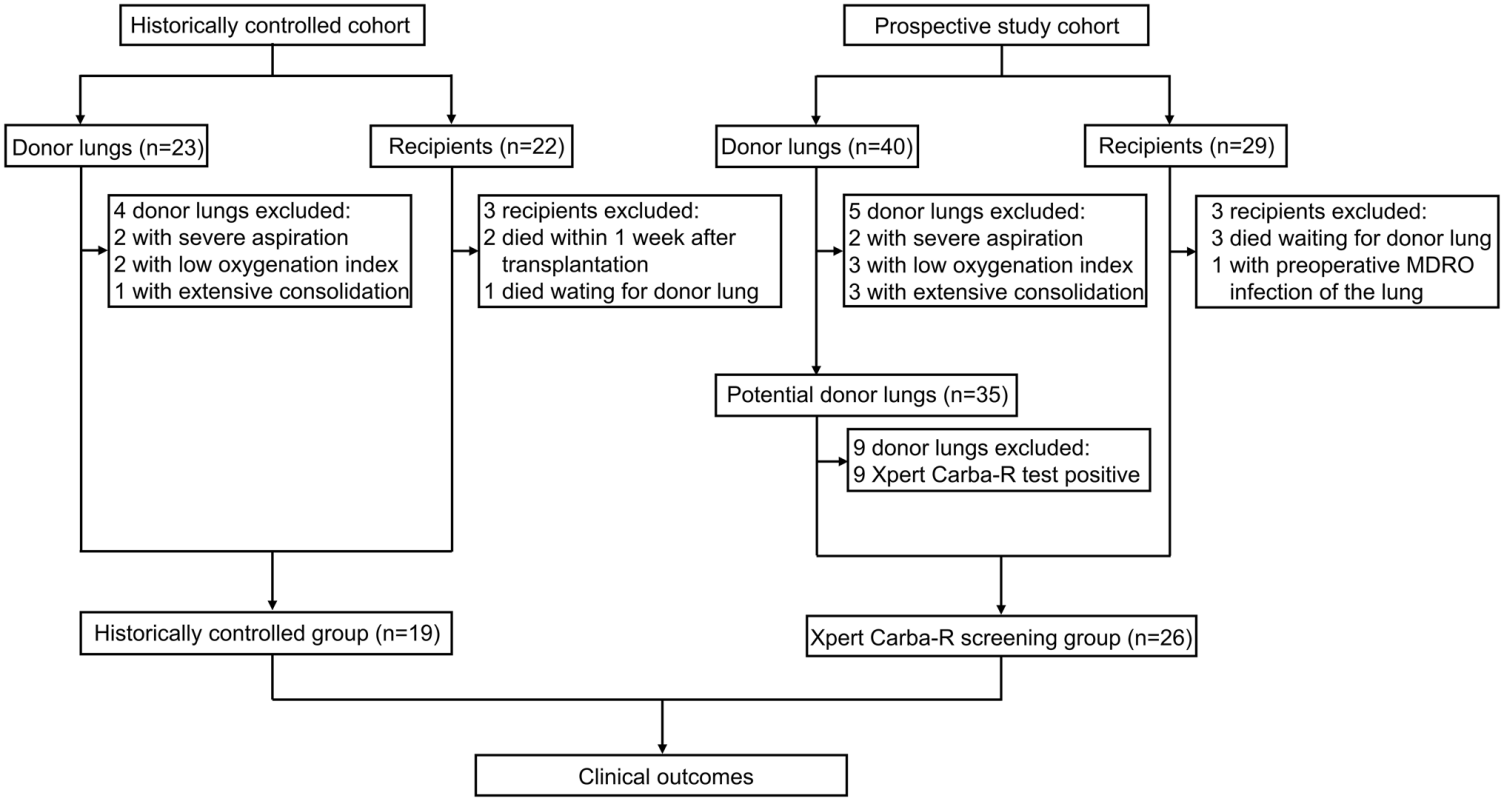
Study flowchart. MDRO, multidrug-resistant organisms

The study design is shown in Fig. 1. From November 2021 to September 2022, twenty-nine consecutive recipients were observed in the study. Among them, three cases died during the waiting period for donor lung supply, and one of these cases had an MDRO infection in the lung. The remaining twenty-six recipients eligible for inclusion criteria were included in the Xpert Carba-R screening group. During the same period, forty donor lungs were available for transplant. Thirty-five donor lungs met the inclusion criteria and five dropped out for various reasons. The thirty-five donor lungs were defined as potential donor lungs for Xpert Carba-R testing. If one or more carbapenemase gene (including *bla*_*KPC*_*, bla*_*NDM*_*, bla*_*VIM*_*, bla*_*IMP*_, and *bla*_*OXA-48*_) was positive in BALF by Xpert Carba-R, the potential donor lungs were not acquired and would be declined for the subsequent lung transplantation. Only the potential donor lungs of carbapenemase gene negative were used as donor lungs in the Xpert Carba-R screening group. For the historic controls, twenty-three cases of donor lungs and twenty-two recipients were consecutively observed from January 2016 to December 2020. According to the inclusion criteria, nineteen recipients and the matching donor lungs were included.

### Donor inclusion criteria

Donor meeting the following criteria were considered for inclusion in the study: legal and ethical instruments of organ donation can be obtained; DBD; same blood type or different but compatible with recipient blood type; age ≥ 18 and ≤ 70 years; oxygenation index (P/F) > 230 mmHg (FiO_2_ = 1.0, PEEP = 5 cm H_2_O); chest radiograph showing a clear lung field or a mild to moderate exudation; appropriate size-match in lung donor and recipient, or a poor size-match but with satisfactory matching after volume reduction to donor lung; absence of chest trauma; absence of aspiration or slight aspiration improved after treatment; absence or a small amount of purulent secretions in the airway; cold ischemia time < 8 h; and no evidence of MDRO or CRO infection and colonization.

### Recipient inclusion criteria

The patients who suffered non-neoplastic pulmonary disease accepted for lung transplantation showed: normal blood sugar levels or fasting blood glucose of diabetic patients that were controlled below 10 mmol/L; normal or mildly abnormal liver function (Child–Pugh grade A); normal or mildly abnormal renal function (GFR > 59 ml/min); age ≤ 75 years; a postoperative survival time was more than 1 week; no evidence of MDRO (including CRO, methicillin-resistant Staphylococcus aureus (MRSA), vancomycin-resistant enterococci (VRE), extended-spectrum β-lactamases (ESBLs)-producing bacteria, or multidrug-resistant tuberculosis (MDR-TB)) infection or colonization were noted before lung transplantation. For MDRO detection, BALF or sputum specimens of recipients were obtained every three days for microbial culture and antimicrobial susceptibility testing before lung transplantation.

### Xpert Carba-R CRO detection in donor lung

For the BALF sample, we used the Xpert Carba-R test with the off-label following procedure: approximately 15 ml of sterile normal saline was injected into the left and right main bronchus of the donor lung by bronchoscopy. At least 20 ml BALF was recovered by aspiration. One ml of the BALF suspension was added to a 5 mL Xpert Carba-R sample reagent vial and mixed for 10 s. Using the supplied pipette, 1.7 ml of sample reagent was transferred into the sample chamber of the Xpert Carba-R cartridge, the lid was closed, and the cartridge was placed in the Cepheid GeneXpert platform for testing.

### Bacterial culture of BALF samples

10 µl of BALF from the donor lung was inoculated onto a Columbia blood agar blood plate, a vancomycin-containing chocolate agar plate, and a crystal violet-containing MacConkey agar plate, and incubated at 35 °C for 24–48 h. When the BALF samples grew more than 10^4^ CFU/mL, colonies were selected and identified using matrix-assisted laser desorption ionization time-of-flight mass spectrometry (MALDI-TOF–MS). Antimicrobial susceptibility testing (AST) of these identified bacteria was performed by using the disc diffusion method and the broth microdilution method according to the 2022 Clinical Laboratory Standard Institute (CLSI) guidelines [14].

### Clinical outcomes

DDI was defined as any infection present in the donor that is transmitted to one or more recipients during or after transplantation [15]. At present, there is still a lack of effective methods for the identification of DDI in donor infections, especially the lack of consensus on the identification of DDI using molecular diagnostic tests. In this study, BALF or sputum specimens from recipients were obtained every three days for microbial culture and antimicrobial susceptibility testing before lung transplantation. Cultures growing MDRO, especially those indicating CRO -infected or colonized- recipients, were used to exclude those patients from the study cohort. Therefore, in this study, we defined CRO-associated DDI as an infection in a recipient within 1 week after lung transplantation, if the CRO-containing organism recovered from the recipient is the same as the CRO-containing organism carried by the donor lung and the organisms has the same antimicrobial susceptibility profile. Death of recipients related by infection with any donor- or recipient-derived pathogen within 60 days after lung transplant was described as infection-related death within 60 days, which includes CRO-associated DDI-related death within 60 days.

In this study, we collected the clinical characteristics of the donors and recipients to compare the baselines between the two groups. The outcomes and incidence of DDI, CRO-associated DDI, and infection-or CRO-associated DDI-related death within 60 days were mainly focused on the effect of Xpert Carba-R donor lung screening on the prognosis of recipients.

### Statistical analysis

We analyzed variable distribution by the D’Agostino-Pearson test. The continuous variables of normal distributions were expressed as mean ± standard deviation, and the continuous variables of non-normal distribution were presented as medians and quartiles. The classification variables were presented as frequencies and percentages. The continuous variables were compared by an independent sample *t*-test or rank-sum test, and the comparison of classification variables was conducted by Fisher's exact test. Logistic regression was used for the univariate analysis and multivariate analysis. Penalized likelihood logistic regression was used when quasi-complete separation happened. A *p*-value < 0.05 was considered statistically significant. SPSS 26.0 software (IBM, Chicago, USA) plus Normaltest software package and Statistical Analysis System (SAS) statistical software package, version 9.4 were used for statistical analysis.

## Results

In the thirty-five potential donors, nine (25.71%) with positive Xpert Carba-R results in BALF were declined for lung transplantation before the acquisition. Finally, the twenty-six recipients and matching CRO-negative donor lungs (74.29%) were included in the Xpert Carba-R screening group.

The baseline characteristics of donors and recipients in both groups were summarized in Table 1. The factors of donors, including age, sex, death mechanism, duration of brain death declaration (BDD) to lung transplantation, mechanical ventilation support time, and cold ischemia time were not statistically significant between the screening and control groups. In the Xpert Carba-R screening group, the donor lungs of carbapenemase gene negative were used for lung transplantation.

**Table 1:** Baseline characteristics of donors and recipients in the Xpert Carba-R screening group and control group

	Xpert Carba-R screening group (*n* = 26)	Control group (*n* = 19)	*P* value
Donor baseline characteristics			
Age (years; mean ± SD)	42.00 ± 8.16	40.26 ± 10.33	0.53
Male gender (*n*; %)	19/26 (73.08%)	14/19 (73.68%)	1.00
Death mechanism (*n*; %)			0.96
Intracranial hemorrhage	12/26 (46.15%)	9/19 (47.37%)	
Trauma	7/26 (267.92%)	6/19 (31.58%)	
Asphyxiation	3/26 (11.54%)	2/19 (10.53%)	
Other	4/26 (15.38%)	2/19 (10.53%)	
BDD to lung transplantation (hours; mean ± SD)	45.42 ± 18.05	51.58 ± 18.48	0.27
Duration of mechanical ventilation support (hours; mean ± SD)	67.35 ± 19.39	65.79 ± 23.79	0.81
Cold ischemia time of donor lungs (minutes; mean ± SD)	381.65 ± 120.86	349.47 ± 123.94	0.39
Pathogens isolation in donor lungs (*n*; %)			
Pathogens isolation positive	14/26 (53.85%)	9/19 (47.37%)	0.77
CRO isolation positive	2/26 (7.69%)	7/19 (36.84%)	0.02
Type of MDRO isolation in donor lungs (*n*; %)			
*Staphylococcus epidermidis* (MRSE)	1/26 (3.85%)	N/A	
*Acinetobacter baumannii* (CRAB)	1/26 (3.85%)	3/19 (15.79%)	
*Pseudomonas aeruginosa* (CRPA)	N/A	1/19 (5.26%)	
*Klebsiella pneumoniae* (CRKP)	1/26 (3.85%)	3/19 (15.79%)	
Recipient baseline characteristics			
Age (years; mean ± SD)	64.35 ± 6.81	56.90 ± 12.36	0.01
Male gender (*n*; %)	23/26 (88.46%)	15/19 (78.95%)	0.43
Blood type (*n*; %)			0.65
A	9/26 (34.62%)	9/19 (47.37%)	
B	10/26 (38.46%)	4/19 (21.05%)	
O	4/26 (15.38%)	3/19 (15.79%)	
AB	3/26 (11.54%)	3/19 (15.79%)	
BMI (kg/m2;; mean ± SD)	21.76 ± 2.74	20.62 ± 2.95	0.19
Smoking (*n*; %)			0.32
Never smoker	5/26 (19.23%)	7/19 (36.84%)	
Ex-smoker	18/26 (69.23%)	9/19 (47.37%)	
Current smoker	3/26 (11.54%)	3/19 (15.79%)	
Primary diseases (*n*; %)			0.28
COPD	7/26 (26.92%)	5/19 (26.32%)	
ILD	18/26 (69.23%)	11/19 (57.89%)	
LAM	N/A	1/19 (5.26%)	
Pneumonoconiosis	1/26 (3.85%)	N/A	
OB	N/A	2/19 (10.53%)	
Comorbidities (*n*; %)	14/26 (53.85%)	7/19 (36.84%)	0.26
Diabetes mellitus	6/26 (23.08%)	3/19 (15.79%)	
Hypertension	6/26 (23.08%)	5/19 (26.32%)	
Coronary heart disease	2/26 (7.69%)	N/A	
Connective tissue disease	1/26 (3.85%)	N/A	
Preoperative oxygen saturation (FiO_2_: 0.21) (%, mean ± SD)	89.00 ± 5.51	87.21 ± 6.35	0.32
Oxygen support pre-transplantation (*n*; %)			0.25
Nasal oxygen	14/26 (53.85%)	5/19 (26.32%)	
OxyMask	5/26 (19.23%)	8/19 (42.11%)	
High-flow oxygen	4/26 (15.38%)	3/19 (15.79%)	
Mechanical ventilation	3/26 (11.54%)	3/19 (15.79%)	
Laboratory test results (mean ± SD)			
Hemoglobin (g/L)	139.54 ± 19.43	136.05 ± 23.32	0.59
Albumin (g/L)	33.50 ± 6.43	34.00 ± 4.41	0.77
Total bilirubin (mol/L)	13.56 ± 6.13	16.71 ± 10.43	0.21
Serum creatinine (umol/L)	60.73 ± 10.27	55.89 ± 16.52	0.23
Days on waitlist (days; mean ± SD)	26.58 ± 21.11	38.53 ± 35.64	0.17
Types of lung transplantation (*n*; %)			0.23
Double lung	14/26 (53.85%)	10/19 (52.63%)	
Single lung	12/26 (46.15%)	7/19 (36.84%)	
Retransplant	N/A	2/19 (10.53%)	
Preoperative pathogens isolation positive in recipient lungs (*n*; %)	3/26 (11.54%)	2/19 (10.53%)	1.00
Postoperative pathogens isolation in recipient lung grafts (*n*; %)			
Pathogens isolation positive	23/26 (88.46%)	19/19 (100%)	0.25
CRO isolation positive	9/26 (34.62%)	15/19 (78.94%)	0.01
Type of MDRO isolation in postoperative recipient lung grafts (*n*; %)			
*Klebsiella pneumoniae* (ESBL-E)	1/26 (4.55%)	N/A	
*Staphylococcus aureus* (MRSA)	N/A	1/19 (5.26%)	
*Acinetobacter baumannii* (CRAB)	4/26 (15.38%)	8/19 (42.11%)	
*Pseudomonas aeruginosa* (CRPA)	4/26 (15.38%)	5/19 (26.32%)	
*Klebsiella pneumoniae* (CRKP)	4/26 (15.38%)	6/19 (31.58%)	
Different classes of antibiotics used (mean ± SD)	5.19 ± 2.58	5.05 ± 2.12	0.85
Ceftazidime/avibactam used (*n*; %)	4/26 (15.38%)	3/19 (15.79%)	1.00

*P* value calculated with *t*-test or Chi-square test as appropriate. *BDD* duration of brain death declaration, *COPD* chronic obstructive pulmonary disease, *ILD* interstitial lung disease, *LAM* lymphangioleiomyomatosis, *OB* obliterative bronchiolitis, *CRO* carbapenem-resistant organisms, *MDRO* multi-drug resistant organisms, *MRSE* methicillin-resistant *Staphylococcus epidermidis*. *ESBL-E* extended-spectrum β-lactamase-producing Enterobacteriaceae, *MRSA* methicillin-resistant *Staphylococcus aureus*, *CRAB* carbapenem-resistant *Acinetobacter baumannii*, *CRPA* carbapenem-resistant *Pseudomonas aeruginosa*, *CRKP* carbapenem-resistant *Klebsiella pneumoniae*

In this study, microbial culture and antimicrobial susceptibility testing of BALF specimens from donor lungs were routinely cultured prior to lung transplantation regardless of the Xpert Carba-R results. The BALF culture reports of donors in the Xpert Carba-R screening group suggested that in addition to one case of methicillin-resistant *Staphylococcus epidermidis* (MRSE) infection, there was one case of carbapenem-resistant *Klebsiella pneumoniae* (CRKP) infection, and one case carbapenem-resistant *Acinetobacter baumannii* (CRAB) infection.

The rate of CRO isolation from donor lung specimens was significantly lower in the Xpert Carba-R screening group (2/26, 7.69%) compared with that in the control group (7/19, 36.84%) (*p* = 0.02) (Table 1). For specimens from recipients, there was a significant difference between the two groups in CRO positivity rate of postoperative lung grafts (9/26, 34.62% vs. 15/19, 78.94%, *p* < 0.01) (Table 1).

Whether the presence of CRO infection in the donor lung has effects on DDI and outcomes of recipients is unclear. In this study, compared with non-CRO infected-donor lungs, the use of donor lungs with CRO infection significantly increased DDI (*p* < 0.01) and infection-related death within 60 days (*p* < 0.01) in recipients after lung transplantation (Table 2).

**Table 2:** The effict of CRO isolation in preoperative donor lungs on outcomes in lung transplant recipients

Outcomes of recipients	CRO isolation positive in donor lungs (*n* = 9)	Non-CRO isolation positive in donor lungs (*n* = 36)	*P* value
DDI (*n*; %)	8/9 (88.89%)	4/36 (11.11%)	< 0.01
Infection-related death within 60 days (*n*; %)	7/9 (77.78%)	2/36 (5.56%)	< 0.01
Duration of postoperative ICU treatment (days; mean ± SD)	36.89 ± 20.05	27.86 ± 27.11	0.36
Postoperative consumption of therapeutic antibiotics (cumulative DDDs, mean ± SD)	94.16 ± 58.80	58.57 ± 60.63	0.12

*DDI* donor-derived infection, *CRO* carbapenem-resistant organisms, *cumulative DDDs* the sum of the DDDs of each therapeutic antibiotic used for postoperative lung transplant recipients, *DDDs* the mass of each antibiotic consumed/ defined daily dose (DDD)

Among forty-five lung transplantation patients (twenty-six in the Xpert Carba-R screening group and nineteen in the control group), DDI and CRO-associated DDI occurred in 19.23% and 7.69% in the screening group, and 36.84% and 31.58% in the control group, respectively (Table 3). We observed several infection-related deaths within 60 days. There were two (7.69%) and seven (36.84%) deaths in the two groups, respectively (OR = 0.14, 95% CI 0.03–0.80, *p* = 0.02), including one CRO-related death due to DDI (3.85%) in the screening group and six (31.58%) in the control group (OR = 0.09, 95% CI 0.01–0.80, *p* = 0.03) (Tables 3 and 4). Adjusted for the potential confounders of BMI and diabetes mellitus in recipients suggested by the results of univariate analysis, multivariate analysis showed that Xpert Carba-R screening for donor lungs significantly reduced the risk of CRO-associated DDI relating death within 60 days in recipients (OR = 0.05, 95% CI 0.003–0.74, *p* = 0.03) (Table 4). In this study, the Xpert Carba-R screening group received a lower dose of therapeutic antibiotics than the control group (*p* = 0.02) (Table 3).

**Table 3:** The effect of Xpert Carba-R screening on outcomes in lung transplant recipients

Outcomes of recipients	Xpert Carba-R screening group (*n* = 26)	Control group (*n* = 19)	*P* value
DDI (*n*; %)	5/26 (19.23%)	7/19 (36.84%)	0.31
CRO-associated DDI (*n*; %)	2/26 (7.69%)	6/19 (31.58%)	0.06
Infection-related death within 60 days (*n*; %)	2/26 (7.69%)	7/19 (36.84%)	0.02
CRO-associated DDI relates death within 60 days (*n*; %)	1/26 (3.85%)	6/19 (31.58%)	0.03
Duration of postoperative ICU treatment (days; mean ± SD)	24.65 ± 16.33	36.53 ± 34.40	0.13
Postoperative consumption of therapeutic antibiotics (cumulative DDDs, mean ± SD)	47.20 ± 44.21	90.99 ± 72.86	0.02

*DDI* donor-derived infection, *CRO* carbapenem-resistant organisms, *cumulative DDDs* the sum of the DDDS of each therapeutic antibiotic used for postoperative lung transplant recipients, *DDDs* the mass of each antibiotic consumed/ defined daily dose (DDD)

**Table 4:** Effect of Xpert Carba-R screening and other clinical characteristics on CRO-associated DDI relates death within 60 days in recipients, univariable and multivariable analyses

Variables	Level	CRO-associated DDI relates death within 60 days		P value (Fisher's exact test)	Univariable analyses			Multivariable analyses		
		Yes	No		*P* value	OR	95%CI	*P* value	OR	95%CI
Sex of recipients	Male	6 (16.22%)	31 (83.78%)	1.00						
	Female	1 (12.50%)	7 (87.50%)		0.79	0.74	0.08–7.15			
Age of recipients	≥ 60	6 (20.00%)	24 (80.00%)	0.40						
	< 60	1 (6.67%)	14 (93.33%)		0.27	0.29	0.03–2.62			
Cold ischemia time (CIT) of donor lungs	Long CIT	5 (20.83%)	19 (79.17%)	0.42						
	Short CIT	2 (9.52%)	19 (90.48%)		0.31	0.40	0.07–2.32			
CRO isolation positive in donor lungs	Yes	7 (77.78%)	2 (22.22%)	< 0.01						
	No	0 (0.00%)	36 (100.00%)		< 0.01	0.01	0.00–0.11			
BMI (kg/m2) of recipients	≤ 18.5	3 (30.00%)	7 (70.00%)	0.17						
	> 18.5	4 (11.43%)	31 (88.57%)		0.17	0.30	0.06–1.66	0.33	0.37	0.05–2.76
Smoking status of recipients	Current smoker	1 (16.67%)	5 (83.33%)	1.00						
	Non-current smoker	6 (15.38%)	33 (84.62%)		0.94	0.91	0.09–9.22			
Primary lung diseases in recipients	COPD	2 (18.18%)	9 (81.82%)	1.00						
	Other	5 (14.71%)	29 (85.29%)		0.78	0.78	0.13–4.70			
Types of lung transplantation	Double-lung transplant	5 (21.74%)	18 (78.26%)	0.41						
	Other	2 (9.09%)	20 (90.91%)		0.26	0.36	0.06–2.09			
Diabetes mellitus in recipients	Yes	3 (33.33%)	6 (66.67%)	0.13						
	No	4 (11.11%)	32 (88.89%)		0.12	0.25	0.04–1.41	0.06	0.09	0.01–1.10
Xpert Carba-R screening for donor lungs	Control group	6 (31.58%)	13 (68.42%)	0.03						
	Xpert Carba-R screening group	1 (3.85%)	25 (96.15%)		0.01	0.09	0.01–0.80	0.03	0.05	0.00–0.74

*COPD* chronic obstructive pulmonary disease, *CRO* carbapenem-resistant organisms, *DDI* donor-derived infection, *BMI* body mass index

^1^Logistic analysis was used for univariate analysis of each factor. While penalized likelihood logistic regression was used for the factor of CRO isolation positive in donor lungs because quasi-complete separation happened

^2^The factors with *P* value < 0.2 (univariate analysis) were added to the multivariate logistic model except the factor of CRO isolation positive in donor lungs for it is a mediator that lies on the causal pathway between the exposure and outcome

^3^Long cold ischemia time was defined as more than mean of cold ischemia time (368 min) in this study

^4^All analyses in this table were conducted using the Statistical Analysis System (SAS) statistical software package, version 9.4

## Discussion

Donor-derived infections, especially those that are CRO-related, are an important risk factor leading to poor outcomes of lung transplantation. Occult CRO infections in donor lungs often result in high mortality, which with carbapenem-resistant *Klebsiella pneumoniae* infection can reach 72.7% [16]. Current CRO screening methods, mainly based on bacterial culture followed by AST, are too slow to provide information on infection in donor lungs before transplantation. Thus, infected lungs may be transplanted, leading to postoperative infections requiring antimicrobial therapy. In addition, CRO-related infections, especially those caused by metallo-β-lactamases (MBLs) producing organisms, are very difficult to treat, leading to higher mortality rates after transplantation. The Xpert Carba-R test, which provides qualitative detection and differentiation of five commonly encountered carbapenemase gene families with a turnaround time of less than one hour, can provide real-time screening for donor organs.

The Xpert Carba-R test has been reported to show excellent performance detecting CRO in a variety of clinical specimens, including 95% sensitivity and 99% specificity for rectal swabs [17], 92.9% sensitivity, and 86.7% specificity for sputum samples [18], and 95% sensitivity and 95% specificity for bronchial aspirates [19], although the latter two specimen types are considered off-label. In this study, the lung acquisition team was able to bring the GeneXpert II instrument and cartridges to the OPO site to enable rapid, point-of-care testing for CRO in bronchoalveolar lavage specimens taken from the donors. This enabled clinicians to recognize CRO infection in the donor lung before acquisition and transplantation, improving the postoperative prognosis of recipients.

In this study, cumulative DDDs were significantly different among patients in the Xpert Carba-R screening group and those in the Control group. There may be a cost-effect in the screening process using the Xpert Carba-R test, although this will require additional studies for confirmation. CRO infections have become a major international public health problem due to inadequate treatment options and the historically slow pace of developing novel antimicrobial drugs. In China, there has been a sharp increase in infections caused by CRO and the emergence of new resistance genotypes in multiple bacterial species has been observed. Some bacterial species have been documented to carry two or more carbapenemase genes [13]. Due to the poor outcome associated with transplanting CRO-positive organs, it is critical to screen organs from DBD and DCD before transplantation. The quick, on-demand, real-time capacity of the Xpert Carba-R test provides enough time for the acquisition team to screen potential donor lungs before transplantation, which is not possible with traditional culture and AST methods. This enables the securing of donor organs more cost-effectively.

To our knowledge, this is the first study demonstrating that accurate screening for CRO in donor lungs in a real-time manner raises the potential to reduce CRO-associated DDI mortality. However, there are several limitations to this study. First, this study used a group of historical patients as controls when the real-time molecular screening method was not yet available. These historical controls may have given results that could be biased by a variety of confounding factors. Such as the low average age of recipients in historical controls. Second, the study was performed in a single center with relatively small numbers of patients recruited. A larger, multi-center clinical trial is being planned with a focus on healthcare economics.

In conclusion, the capacity for quick and accurate detection and characterization of CRO by Xpert Carba-R provides an ideal screening tool in donor lungs before transplantation. This enabled clinicians to know the CRO infection status of donor lungs in a real-time manner and to formulate a more precise plan for acquiring the lung from the donor for transplantation.

## Data Availability

Not applicable.

## References

[1] Hayes D Jr, Harhay MO, Cherikh WS, Chambers DC, Perch M, Khush KK, Hsich E, Potena L, Sadavarte A, Booker S, Singh TP, Zuckermann A, Stehlik J. International Society for Heart and Lung Transplantation. The International Thoracic Organ Transplant Registry of the International Society for Heart and Lung Transplantation: Twenty-fourth pediatric lung transplantation report - 2021; Focus on recipient characteristics. J Heart Lung Transplant. 2021; doi: 10.1016/j.healun.2021.07.018 10.1016/j.healun.2021.07.018 PMC10281818 34561022

[2] Chambers DC, Zuckermann A, Cherikh WS, Harhay MO, Hayes D Jr, Hsich E, Khush KK, Potena L, Sadavarte A, Singh TP, Stehlik J; International Society for Heart and Lung Transplantation. The International Thoracic Organ Transplant Registry of the International Society for Heart and Lung Transplantation: 37th adult lung transplantation report - 2020; focus on deceased donor characteristics. J Heart Lung Transplant. 2020; 10.1016/j.healun.2020.07.009 10.1016/j.healun.2020.07.009 PMC7737221 32782073

[3] Fishman JA, Grossi PA (2014). Donor-derived infection–the challenge for transplant safety. Nat Rev Nephrol.

[4] Wu TJ, Lee CF, Chou HS, Yu MC, Lee WC (2008). Suspect the donor with potential infection in the adult deceased donor liver transplantation. Transplant Proc.

[5] Kaul DR, Vece G, Blumberg E, La Hoz RM, Ison MG, Green M, Pruett T, Nalesnik MA, Tlusty SM, Wilk AR, Wolfe CR, Michaels MG (2021). Ten years of donor-derived disease: a report of the disease transmission advisory committee. Am J Transplant.

[6] Pouch SM, Ison MG (2022). Deceased donors with multidrug-resistant organisms: implications and future directions. Curr Opin Organ Transplant.

[7] Procaccio F, Masiero L, Vespasiano F, Grossi PA, Gagliotti C, Pantosti A, Caprio M, Lombardini L, Nanni CA (2020). Donor-Recipient Infection (DRIn) Collaborative Study Group Organ donor screening for carbapenem-resistant gram-negative bacteria in Italian intensive care units: the DRIn study. Am J Transplant.

[8] Anesi JA, Blumberg EA, Han JH, Lee DH, Clauss H, Climaco A, Hasz R, Molnar E, Alimenti D, West S, Bilker WB, Tolomeo P, Lautenbach E (2019). CDC Prevention Epicenters Program. Risk factors for multidrug-resistant organisms among deceased organ donors. Am J Transplant.

[9] Anesi JA, Blumberg EA, Han JH, Lee DH, Clauss H, Hasz R, Molnar E, Alimenti D, Motzer AR, West S, Bilker WB, Tolomeo P, Lautenbach E (2022). CDC Prevention Epicenters Program Impact of donor multidrug-resistant organisms on solid organ transplant recipient outcomes. Transpl Infect Dis..

[10] Lewis JD, Sifri CD (2016). Multidrug-resistant bacterial donor-derived infections in solid organ transplantation. Curr Infect Dis Rep.

[11] Mularoni A, Bertani A, Vizzini G, Gona F, Campanella M, Spada M, Gruttadauria S, Vitulo P, Conaldi P, Luca A, Gridelli B, Grossi P (2015). Outcome of transplantation using organs from donors infected or colonized with carbapenem-resistant gram-negative bacteria. Am J Transplant.

[12] Zhou M, Wang D, Kudinha T, Yang Q, Yu S, Xu YC (2018). Comparative evaluation of four phenotypic methods for detection of class a and b carbapenemase-producing enterobacteriaceae in China. J Clin Microbiol..

[13] Liu Z, Bai L, Liu J, Lei J, Gao X, Tenover FC, Lei K, Tang YW, Geng Y, He A (2021). Parallel validation of the NG-test carba 5 and the Xpert Carba-R for detection and characterization of carbapenem-resistant enterobacterales causing bloodstream infections. J Mol Diagn.

[14] James S. Lewis II, Melvin P. Weinstein, April M. Bobenchik, et al. M100 Performance Standards for Antimicrobial Susceptibility Testing, 32nd Edition 2022.

[15] Wolfe CR, Ison MG (2019). AST Infectious Diseases Community of Practice donor-derived infections: guidelines from the American society of transplantation infectious diseases community of practice. Clin Transplant.

[16] Raviv Y, Shitrit D, Amital A, Fox B, Bakal I, Tauber R, Bishara J, Kramer MR (2012). Multidrug-resistant Klebsiella pneumoniae acquisition in lung transplant recipients. Clin Transplant.

[17] Bai Y, Hao Y, Shao C, Wang Y, Jin Y (2021). Accuracy of Xpert Carba-R assay for the diagnosis of carbapenemase-producing organisms from rectal swabs and clinical isolates: a meta-analysis of diagnostic studies. J Mol Diagn..

[18] Cai Z, Tao J, Jia T, Fu H, Zhang X, Zhao M, Du H, Yu H, Shan B, Huang B, Chen L, Tang YW, Jia W, Qu F (2020). Multicenter evaluation of the Xpert Carba-R assay for detection and identification of carbapenemase genes in sputum specimens. J Clin Microbiol.

[19] Burillo A, Marín M, Cercenado E, Ruiz-Carrascoso G, Pérez-Granda MJ, Oteo J, Bouza E (2016). Evaluation of the Xpert Carba-R (Cepheid) Assay Using Contrived Bronchial Specimens from Patients with Suspicion of Ventilator-Associated Pneumonia for the Detection of Prevalent Carbapenemases. PLoS ONE.

